# A novel protocol for the preparation of active recombinant human pancreatic lipase from *Escherichia coli*

**DOI:** 10.1093/jb/mvy067

**Published:** 2018-08-08

**Authors:** Nanami Kawaguchi, Kimie Date, Yusuke Suzuki, Chihiro Tomita, Rina Naradate, Tomoko Higami, Kosuke Nakamura, Kyoko Aikawa, Haruko Ogawa

**Affiliations:** 1 Chemistry and Biochemistry, Division of Advanced Sciences, Graduate School of Humanities and Sciences; 2 Glycoscience Division, Institute for Human Life Innovation, Ochanomizu University, 2-1-1 Otsuka, Bunkyo-ku, Tokyo, Japan; 3 Department of Materials and Applied Chemistry, College of Science and Technology, Nihon University, 1-5-1 Kanda Surugadai, Chiyoda-ku, Tokyo, Japan

**Keywords:** *Escherichia coli*, human pancreatic lipase, lipolytic activity, refolding, Strep-tag II

## Abstract

An active recombinant human pancreatic lipase (recHPL) was successfully prepared for the first time from the *Escherichia coli* expression system using short Strep-tag II (ST II). The recHPL–ST II was solubilized using 8 M urea from *E.coli* lysate and purified on a Strep-Tactin-Sepharose column. After refolding by stepwise dialyses in the presence of glycerol and Ca^2+^ for 2 days followed by gel filtration, 1.8–6 mg of active recHPL–ST II was obtained from 1 L of culture. The recHPL was non-glycosylated, but showed almost equal specific activity, pH-dependency and time-dependent stability compared to those of native porcine pancreatic lipase (PPL) at 37°C. However, the recHPL lost its lipolytic activity above 50°C, showing a lower heat-stability than that of native PPL, which retained half its activity at this temperature.

Overweight and obesity are considered risk factors for several chronic diseases and disorders. In 2014, more than 1.9 billion adults, 18 years and older, were estimated to be overweight. Of these, over 600 million have been classified as obese by the World Health Organization (WHO), amounting to 11% and 15% of all men and women, respectively. In addition, 41 million children under the age of 5 were overweight or obese in 2014 (1), indicating that a very large number of people are suffering from obesity worldwide. Overweight and obesity are mainly caused by the excess ingestion of lipids and are associated with a higher risk of major lifestyle-related diseases (2). Since triglycerides constitute 90% of dietary fat, pancreatic lipase (triacylglycerol hydrolase; EC 3.1.1.3) is responsible for the digestion of 40–60% of the total ingested fat (3). The control of lipase activity is therefore one of the avenues to improve worldwide healthcare for overweight and obese patients, and the importance of elucidating the properties of pancreatic lipase to control the *in vivo* activity of this enzyme is crucial. Patients with lipase-deficiency or shortage are currently treated with native porcine pancreatic lipase (PPL). However, this treatment cannot be used for patients with pig allergies or who have religious objections to consuming pork, making the use of recombinant human pancreatic lipase (recHPL) inevitable as an effective supplement.

Human pancreatic lipase (HPL) is the main lipolytic enzyme involved in the digestion of dietary fat and represents 3% of the total protein secreted by the exocrine pancreas (4, 5). HPL is a 50-kDa glycoprotein, 449 amino-acid long polypeptide (4, 6), composed of two domains and an *N*-linked glycan on Asn^167^ similar to PPL (4, 5, 7). HPL is synthesized and secreted in its mature form. The three-dimensional structure of HPL revealed the presence of a catalytic triad (Ser^153^–Asp^177^–His^264^) in the active site covered by a ‘lid’ region formed by the large N-terminal domain of the protein. The mechanism of lipid hydrolysis by this enzyme has been elucidated. In the closed conformation, the lid prevents access of substrate to the active site, while in the presence of a lipid–water interface, the lid is displaced to one side, exposing both the active site and a larger hydrophobic surface that allows hydrolysis of the substrate. The open conformation, being the active conformation of HPL, is stabilized upon complexation with colipase through the smaller C-terminal domain of the former (8–10).

HPL preparations of constant and reproducible activity are needed as references to improve the accuracy of measurements of lipase activity in clinical samples, to permit assessment of new therapeutic methods and facilitate comparison of methods between different laboratories. However, obtaining pure and stable native mammalian lipase has been a challenge since it is often degraded by co-existing proteases. Because of the limited availability of human tissue samples and their potential for disease transmission, it may be best to prepare HPL using recombinant expression systems. Here we report the expression, purification, optimized refolding procedures and preliminary characterization of active recHPL from *Escherichia coli*, thus establishing it as a suitable system for the production of recHPL of high purity in large quantities.

## Materials and Methods

### Materials

Ampicillin was obtained from Wako Pure Chemical Industries Ltd (Osaka, Japan). Anhydrotetracycline and Strep-Tactin-Sepharose were purchased from IBA. Triton X-100, ethylenediaminetetraacetic acid (EDTA), urea, l-arginine hydrochloride, l-cystine dihydrochloride, l-cysteine, bovine serum albumin (BSA), Tween 20 and 5, 5′-dithiobis-2-nitrobenzoic acid (DTNB) were obtained from Wako Pure Chemical Industries Ltd. Desthiobiotin and Orlistat were purchased from Sigma-Aldrich (Saint Louis, MO). Phenylmethylsulfonyl fluoride (PMSF) was purchased from Nacalai Tesque, Inc. (Kyoto, Japan). Rabbit anti-HPL polyclonal IgGs and anti-Strep-tag II (ST II) monoclonal IgG were obtained from Abcam (Cambridge, UK) and IBA GmbH (Gottingen, Germany), respectively. Horseradish peroxidase (HRP)-conjugated goat anti-rabbit IgG and anti-mouse IgG antibodies were from Kirkegaard and Perry Laboratories (Washington, DC, USA). Tributyrin was purchased from Wako Pure Chemical Industries Ltd (Osaka, Japan). Porcine colipase was from AbD Serotec (Kidlington, UK). Gel-filtraion FPLC columns, HiPrep 16/60 Sephacryl S-200 HR were purchased from GE Healthcare.

### Construction of HPL expression plasmid

The coding sequence of *HPL* was amplified using human pancreas cDNA library, Human MTC Panel I (Clontech, Laboratories, Inc., Mountain View, CA, USA) as the template for PCR using Primer 1, 5′-AAAGAAGTTTGCTACGAAAGACTC-3′, and Primer 2,5′-GATTGTGCCACACTCCCACTCG-3′. *HPL* amplicon (1,350 bp) was confirmed by agarose electrophoresis. To express ST II-tagged HPL, a second PCR was performed using primers P5, 5′-ATGGTAGGTCTCAAATGAAAGAAGTTTGCTACGAAAGACTCG-3′, and P2, 5′-ATGGTAGGTCTCAGCGCTACACGGTGTGAGGGTGAGCAG-3′. The amplicon was digested using *Bsa*I and ligated with the pASK-IBA3plus plasmid (IBA GmbH) to obtain an HPL-pASK-IBA3plus plasmid. Each PCR was carried out by the manufacturer’s instruction; in a reaction mixture (final volume = 50 μl) containing 5 μl 10 × PCR buffer (Toyobo Co. Ltd, Osaka, Japan), 0.2 mM dNTP, 1 mM SO_4_, 0.3 µM forward and reverse primers, 0.8U KOD plus DNA polymerase (Toyobo Co. Ltd) and 25 ng template DNA. The PCR conditions were as follows: 94°C for 10 min, followed by 25 cycles of denaturation at 94°C for 15 s, annealing at 65°C for 30 s, extension at 68°C for 60 s and terminal elongation at 72°C for 7 min. PCRs were carried out using a ZYMOREACTOR II (ATTO) thermal cycler.

All the constructed plasmids were sequenced using Prism 3100 Avant sequencer (Applied Biosystems, Foster City, CA, USA), and the sequences of the designed recombinant HPLs were confirmed (see Supplementary Fig. S1).

### Expression of ST II-fused recHPL

The *E.coli* DH5α transformants harbouring HPL-pASK-IBA3plus were grown in 5 ml LB medium (+100 μg/ml ampicillin) at 37°C for 18 h. The bacterial suspension (3 ml) was transferred into 300 ml each of fresh LB medium (+100 μg/ml ampicillin) and cultured for 3 h at 37°C (150 rpm).When the absorbance at 550 nm of the culture reached 0.5, expression of recombinant protein was induced by adding anhydrotetracycline at a final concentration of 200 μg/ml. After incubation for 5 h at 30°C with shaking at 120 rpm, cells were harvested by centrifugation at 6,000 rpm (6,100 × *g*) for 15 min.

### Isolation of inclusion bodies and purification of recHPL

The bacterial pellet obtained after induction was resuspended in 15 ml of lysis buffer: buffer A (10 mM Tris-HCl (pH 8.0), 150 mM NaCl, 1 mM EDTA) and frozen at −80°C until further use. The cells were thawed and lysed by sonication. The cellular debris was collected by centrifugation at 6,000 rpm (4,400 × *g*) for 15 min and the precipitate was washed once with 15 ml of the lysis buffer containing 0.5% Triton X-100 and twice with 30 ml of buffer A, followed by centrifugation at 15,000 rpm (28,000 × *g*) for 10 min. The washed inclusion bodies were solubilized with 10 ml of buffer A which contained 8 M urea and 1 mM PMSF (buffer A*) with agitation for 30 min at 4°C. Following centrifugation at 15,000 rpm (28,000 × *g*) for 10 min, the supernatant was collected and dithiothreitol was added at a final concentration of 10 mM. The sample was incubated for 1 h at 37°C with agitation and then dialyzed against equivalent buffer (10 mM Tris-HCl (pH 8.0), 150 mM NaCl, 400 mM l-Arg, 1 mM EDTA, 1 mM PMSF) at 4°C for 2 h to remove urea. The sample and 2 ml of Strep-Tactin-Sepharose (11, 12) were mixed together and incubated at 4°C for 2 h, following which the mixture was packed into a chromatography column. The column was washed with 20 ml equivalent buffer. ST II-fused recHPL was eluted with equivalent buffer containing 2.5 mM desthiobiotin (13). The peak fraction was collected and stored at −80°C. The purity and yield of the protein were analysed by 12% SDS-PAGE and the protein concentration was determined by Tonein-TP assay (Otsuka Pharmaceutical Factory, Inc., Tokyo Japan) using BSA as protein standard.

### Refolding of recHPL

**Scheme 1:**
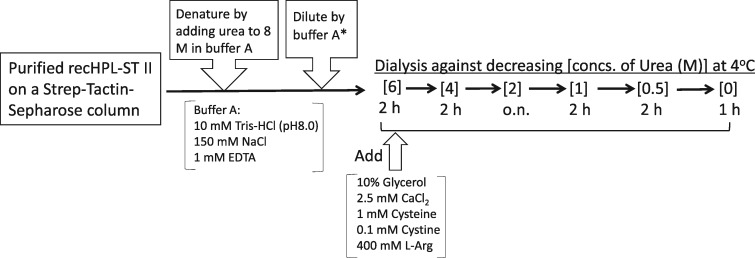
Procedure for preparation of active recHPL–ST II.

The all following processes were performed at 4°C (see Scheme 1), because the yield of the recHPL after refolding was higher when it was refolded at 4°C than that at 25°C though the specific activities of the recHPLs did not significantly different between the two temperatures. For refolding recHPL, the urea-denatured protein was diluted to 0.1–0.2 mg/ml with buffer A* and then dialyzed against 300 ml of refolding buffer (10 mM Tris-HCl (pH 8.0), 150 mM NaCl, 10% glycerol, 2.5 mM CaCl_2_, 1 mM PMSF, 1 mM cysteine, 0.1 mM cystine, 400 mM l-Arg, 6 M urea) with gentle stirring for 2 h. The concentration of urea in the dialysis buffer was then gradually decreased to 4 M over 2 h, followed by a step-wise decrease in urea concentration (2, 1, 0.5 and 0 M) in the dialysis buffer over 2 h for each concentration. Cysteine and cystine were added to promote disulfide linkage formation of recHPL. After urea was completely removed, protein aggregates were removed by centrifugation at 21,000 × *g* for 5 min and the concentration of the refolded protein in the supernatant was measured by Tonein-TP assay using BSA as standard.

### Purification of active recHPL by FPLC

Refolded recHPL (≤2 ml) was injected into a HiPrep 16/60 Sephacryl S-200 HR column (120 ml, GE Healthcare) that had been previously equilibrated with running buffer (10 mM phosphate buffer (pH 8.0), 150 mM NaCl, 400 mM l-Arg). The sample was purified on an Akta Purifier FPLC system (GE Healthcare) at 4°C, using a buffer flow-rate of 0.5 ml/min, and protein peaks were detected by measuring absorbance at 280 nm using a UPC-900 monitor.

### Matrix-assisted laser desorption/ionization time-of-flight mass spectrometry (MALDI-TOFMS)

MALDI-TOFMS (JEOL, Akishima, Japan) was used to measure the molecular weights of recHPL and PPL. MS spectra were calibrated externally with peaks from the protein calibration standard, insulin oxidized B chain ([M + H]^+^, 3, 494.65), and purified bovine serum albumin fraction V ([M + H]^+^, 66, 465.8). The matrix was sinapic acid at a concentration of 10 mg/ml in acetonitrile/water (7:3, v/v). The sample (recHPL and PPL) and matrix were placed on a target plate for crystallization. Crystallization was accelerated by a gentle stream of cold air.

### Measurement of circular dichroism (CD)

CD spectra were measured on a Jasco-820 spectrophotometer (Jasco, Tokyo, Japan) in cells with a 0.2 cm path length. The recHPL and PPL were prepared in 10 mM phosphate buffer (pH 7.5). The spectrum of each sample was measured and recorded in the range of 190–250 nm, with 16 scans conducted for each spectrum. The Principal Component Regression (PCR) method was applied to analyse the secondary structure.

### Dot blot analysis

Three-fold dilution series of purified recHPL and PPL (each 100 μl) were dot-blotted onto polyvinylidene fluoride (PVDF) membranes and blocked with 3% BSA in 10 mM Tris-HCl saline (pH 7.5), then cut into lanes and reacted with the rabbit anti-HPL IgGs for immunoblotting (Abcam, ab96100, 1:1,000) and the HRP labelled anti-rabbit IgG Fc (Millipore, AP156P, 1:5,000) at room temperature for 1 h each. The spots were visualized by ECL reagent (GE Healthcare ECLTM Western Blotting Detection Reagents). Chemiluminescence was detected using an ImageQuant LAS4000 mini imager (GE Healthcare). The plotted signal intensities were calculated using ImageJ (NIH) software.

### Measurement of lipolytic activity (temperature, pH and time dependency, substrate selectivity, inhibitor sensitivity and kinetic studies)

Lipase activity was measured using tributyrin or triolein as substrate by automated titration of fatty acids released with 10 mM NaOH using the pH-stat AT-510 (Kyoto Electronics Manufacturing Co., Ltd) (14). Tributyrin (0.11 M) was emulsified in 1 ml reaction buffer containing 1 mM Tris-HCl (pH 7.5), 4 mM sodium taurodeoxycholate, 100 mM NaCl and 5 mM CaCl_2_. The measurements were performed maintaining colipase in excess, at 0.5 µg/50 µl. After addition of purified enzyme or the fraction eluted by gel filtration FPLC of recHPL (0.5 μg/50 μl), the NaOH consumption was recorded at a reaction temperature of 37°C. One unit was defined as the amount of enzyme that released 1.0 μmol of free fatty acid per min. Heat stability was examined by measuring activity at between 37 and 55°C. The effect of pH on lypolytic activity was measured by changing the pH of the reaction buffer and performing the assay at 37°C. The effect of a pancreatic lipase inhibitor, Orlistat (tetrahydrolipstatin), at 0–25 μg/ml on lipase activity was measured using the standard protocol (15). The lipolytic activity against tributyrin was analysed by the Michaelis–Menten equation and the Michaelis constants (*K*_m_) and the catalytic constants (*k*_cat_) for PPL and recHPL were determined by using the Lineweaver–Burk plot obtained from the initial velocity studies using 2.5 to 30 mM tributyrin as substrate at 37°C.

### SDS-PAGE and western blot analysis

For SDS-PAGE, samples were dissolved in the sample buffer as described by Laemmli (16), containing 5% 2-mercaptoethanol, and heated at 100°C for 5 min. The samples were electrophoresed in 12% polyacrylamide gels and visualized by CBB or silver straining. Proteins were electroblotted onto PVDF membranes, using a semi-dry blotting apparatus. The membrane was blocked with TBS-T buffer (10 mM Tris-HCl buffer (pH 7.5), 0.15 M NaCl, 0.1% Tween-20) containing 3% BSA overnight at 4°C. The membrane was incubated with the primary antibody, namely rabbit polyclonal anti-HPL IgG (1:3,000) at 25°C for 1 h and washed 3 times with TBS-T. The membrane was then incubated with the secondary antibody, HRP-conjugated goat anti-rabbit IgGs (1:5,000) for anti-pancreatic lipase polyclonal antibodies, or anti-ST II polyclonal antibodies at room temperature for 1 h followed by washing 3 times with TBS-T. The bound antibodies were developed using an ECL kit (GE Healthcare UK Ltd, Buckinghamshire, UK) and analysed on a LAS-3000 (Fujifilm Co., Tokyo, Japan).

### Quantification of free thiol groups using Ellman reagent

The number of free thiol groups in recHPL and native PPL was determined using 5,5′-dithiobis-2-nitrobenzoic acid (DTNB) reagent (17). Samples (300 μl) at 0.0–6.2 μM were dissolved in 10 mM Tris-HCl (pH 8.0), 10 μl of DTNB reagent was added and the absorption at 412 nm was measured every 5 min for 2 h at room temperature. Dithiothreitol (DTT) and l-cysteine containing two and one free thiol groups, respectively, were used as standards. Two well-characterized proteins, bovine beta-lactoglobulin (BbL) and bovine serum albumin (BSA), containing two and one free thiol groups per protein molecule, respectively, were also examined to ensure the reliability of the measurement.

## Results

### Expression and purification of recHPL–ST II

**Fig. 1:**
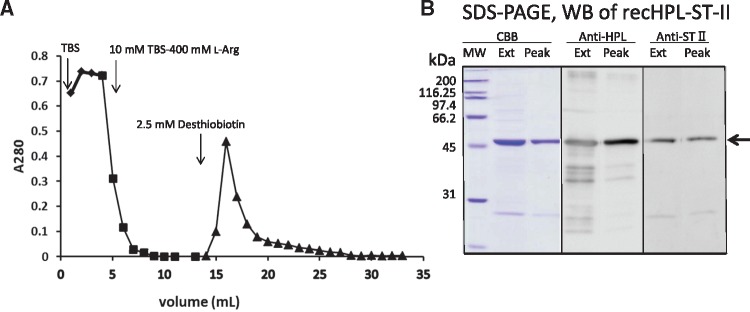
**Purification of recHPL–ST II using Strep-Tactin-Sepharose.** (**A**) Affinity chromatography profile on a Strep-Tactin-Sepharose column. Elution was started from the fraction indicated by the arrow and the peak was obtained at fraction no. 17. (**B**) SDS-PAGE and western blot analysis of the recHPL–ST II. From left lanes, ‘CBB’, CBB-stained 12% polyacrylamide gel showing MW, marker proteins; Ext, solubilized and refolded fusion protein from inclusion body; Peak, the purified recHPL–ST II on the Strep-Tactin-Sepharose column. ‘Anti-HPL’, the fractions Ext and Peak were electroblotted to a PVDF membrane and stained using anti-lipase antibodies and the colour was developed using ECL as described in the text; ‘Anti-ST II’, the fractions Ext and Peak were electroblotted to a PVDF membrane and stained using anti-ST II antibodies and the colour was developed using ECL as described in the text.

Since recHPL–ST II localized to inclusion bodies when expressed in *E.coli* upon induction with anhydrotetracycline, we compared the solubilization efficiency of recHPL–ST II from *E.coli* lysates, using various buffer combinations. We found that between TBS only and TBS supplemented with 8 M urea, 1% Triton X-100, or 1% SDS, TBS containing 8 M urea was most efficient at solubilizing recHPL–ST II (see Supplementary Fig. S2). Therefore, denaturing buffer A*, which contained 8 M urea, was used to solublize recHPL–ST II for further characterization. To improve the extraction efficiency of the recHPL–ST II, the cell pellets were re-extracted by buffer A as described in ‘Materials and Methods’ section and the combined supernatants were applied onto a Strep-Tactin-Sepharose column for purification. As shown in Fig. 1A, the recHPL–ST II eluted from the column as a single peak using desthiobiotin. Both the extract and the eluted peak fraction (15-20 mL) showed a major protein band at around 50 kDa upon SDS-PAGE and CBB staining. Fragments of recHPL–ST II were removed by the Strep-Tactin affinity chromatography (Fig. 1B), as seen by western blot analysis using anti-human pancreatic lipase (HPL) antibodies and anti-ST II antibodies (Fig. 1B). Using our extraction procedure, 1.8–6 mg of pure recHPL was obtained from 1 L of *E.coli* culture.

### Effect of additives on the refolding of recHPL–ST II

**Fig. 2:**
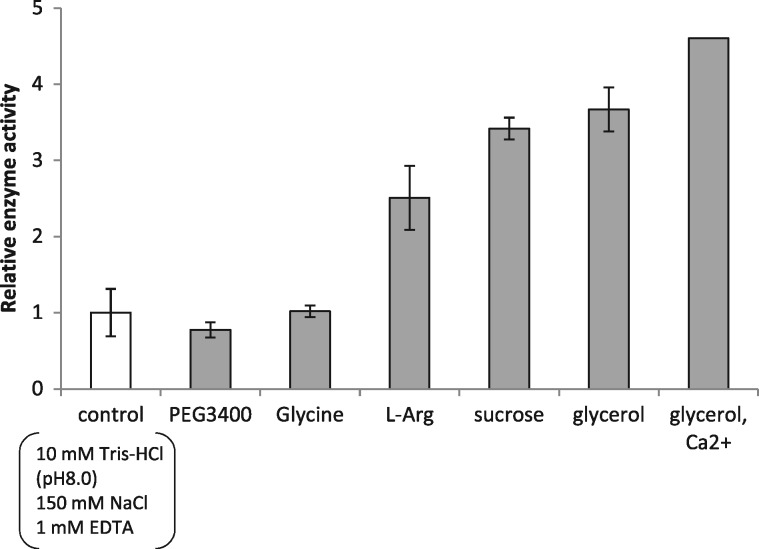
**Effect of additives on the refolding efficiency of recHPL–ST II.** Different additives were added into the refolding buffer, namely, 0.5% PEG3400, 1 M glycine, 400 mM l-Arg, 0.75 M sucrose, 10% glycerol and 10% glycerol + 2.5 mM Ca^2+^. Refolding recovery yield was measured relative to the control refolding condition, obtained without additives as standards (*n* = 3).

The refolding efficiency of the purified recHPL–ST II to the active enzyme was measured by step-wise dialysis in the presence of various additives and the lipolytic activity of the refolded recHPL–ST II. Among the various additives tested, 10% glycerol and 2.5 mM Ca^2+^, 0.75 M sucrose and 0.4 M l-Arg accelerated the refolding of recHPL–ST II more than 2-fold during the removal of urea. As shown in Fig. 2, copresence of 10% glycerol and 2.5 mM Ca^2+^ showed the highest efficiency of refolding, which was 4.6-fold higher than that of control which had been refolded in buffer A and in the absence of additives. Interestingly, the addition of l-Arg showed a remarkable effect on the refolding of recHPL–ST II, resulting in the high yield, which may be attributed to the inhibition of aggregation of recHPL–ST II by l-Arg (18).

### Separation of active recHPL–ST II from inactive fraction

**Fig. 3:**
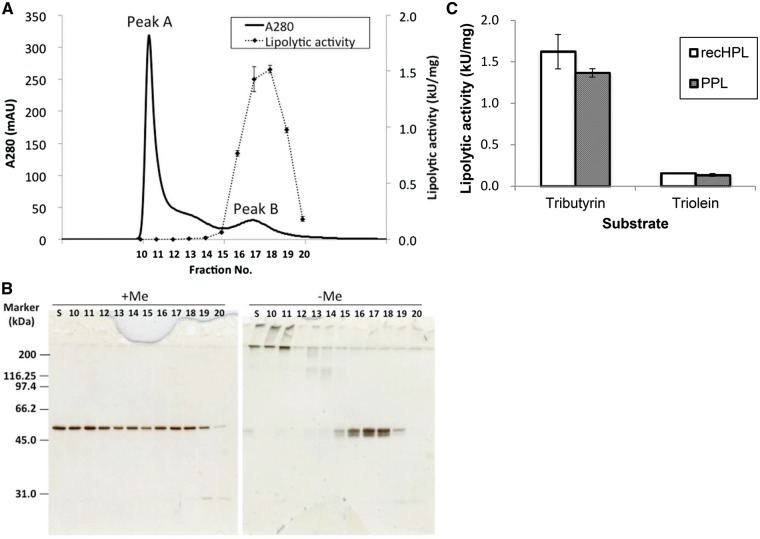
**Purification of active recHPL by gel-filtration FPLC.** (**A**) Elution profile of recHPL on gel filtration. RecHPL after refolding was loaded onto a HiPrep 16/60 Sephacryl S-200 column (column volume: 120 ml) and eluted using 25 mM PBS containing 400 mM l-Arg at a flow rate of 0.5 ml/min at 4°C. Specific activity of the fractions was measured using tributyrin as substrate. (**B**) SDS-PAGE of the fractions eluted from gel filtration chromatography under reducing and non-reducing conditions. Me: 2-mercaptoethanol, Lane S: recHPL after refolding. Lanes numbered as 10–20: correspond to the eluted fractions from No. 10 to 20 in (A), respectively. (**C**) Specific activity of the recHPL that has the highest activity in the eluted fractions and native PPL using tributyrin or triolein as substrates. Data shown represent the mean ± SD values from at least three measurements.

After refolding in the presence of glycerol and Ca^2+^, the recHPL sample was separated into two major peaks by gel-filtration FPLC using a HiPrep 16/60 Sepharcryl S-200 column, as shown in Fig. 3A. The first eluted peak (Peak A) was found to contain inactive aggregates of recHPL–ST II, while the second peak (Peak B) contained the active monomer as indicated by lipolytic activity (Fig 3A) and SDS-PAGE under non-reducing condition and western blot analysis (Fig. 3B, −ME). Under reducing condition in the presence of 2-mercaptoethanol, the fractions of Peak A were completely dissociated to monomer (Fig. 3B, +ME) indicating that the inactive aggregates are stabilized with disulfide linkages.

The results show that the active recHPL expressed using *E.coli* has a specific activity of 1.2–1.9 kU/mg for tributyrin and 0.16 kU/mg for triolein in Peak B, which is almost equal to the activity of natural PPL (1.3–1.9 kU/mg for tributyrin and 0.14–0.15 kU/mg for triolein), as shown in Fig. 3C. Under non-reducing conditions, two bands around 50 kDa in the fractions of Peak B indicated that the monomer recHPL had two forms with different disulfide bridges (Fig. 3B, −ME); therefore, we investigated whether the number of free thiols of recHPL in Peak B corresponded to that of native PPL or not using the Ellman reagent (see Supplementary Fig. S3). It has been known that native PPL and HPL have two free thiol groups and that only one of them can react with the Ellman reagent (19). The number of free thiol group in PPL and recHPL was very close to those of l-cysteine and BSA, -SH=1, indicating that both PPL and recHPL contain one free thiol group in the molecule that can react with the reagent. From these results, we confirmed that recHPL and native PPL have similar thiol redox states and exhibit almost equal specific activities.

### MALDI-TOFMS analyses of recHPL

The total mass number of recHPL and PPL was detected at *m*/*z* 50,858 for recHPL and at *m*/*z* 51,403 for PPL by MALDI-TOFMS. The theoretical peptide masses that were calculated from the amino acid sequences were 49,488 Da for recHPL and 49,824 Da for PPL. The gap between the measured mass number and the calculated one for recHPL was +1,370 Da, which is attributable to the sum of the ST-tag linked via a 2-amino-acid-linker (total 1,215.6 Da) to the C-terminal of recHPL and the Met (149.2 Da) at the N-terminal modification. However, the gap for PPL was +1,579 Da, which is attributable to the N-glycan present at Asn^167^ (Gal_0–2_GlcNAc_4_Man_3_Fuc_1_, the MW of which is totally 1,786–1,462 Da), that has been reported to be most abundant in PPL by Fournet *et al.* (20).

The measured masses for recHPL and PPL coincided with the theoretical values predicted from each primary sequence, and the glycan components reported for PPL. These results support the accuracy of the recHPL sequence and therefore its similarity to PPL in the peptide moiety.

### Characterization of the structures of recHPL and PPL

**Fig. 4:**
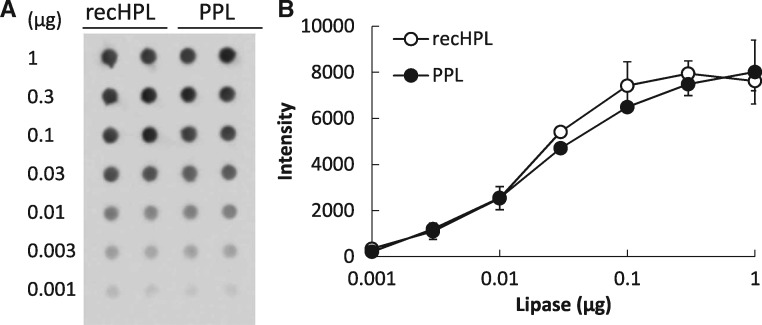
**The reactivity of the recHPL and PPL against the anti-HPL antibodies by dot blot analysis.** (**A**) Dot blot analysis on PVDF membrane stained with the rabbit anti-HPL IgGs for immunoblotting and the HRP-labelled anti-rabbit IgG Fc. (**B**) Densitometric analysis of spots on blotting membrane. Data shown represent the mean ± SD.

Similarity of the structures of the recHPL and the native PPL was studied by CD spectroscopy and immunoreactivity with anti-pancreatic lipase antibodies. The CD spectra of the recHPL and PPL indicated that they contain similar compositions of the secondary structures, which are expressed as % of α-helix:β-sheet:β-turn:other structures, 17:33:13:37 for recHPL, and 15:32:14:40 for PPL. In addition, both lipases were equally recognized by the polyclonal rabbit anti-HPL antibodies in a concentration-dependent manner (Fig. 4), demonstrating that recHPL and PPL share common epitopes between the molecules. These results indicate the close similarity of the structures of the recHPL and PPL.

### Kinetics assays of recHPL and PPL

The kinetics constants of recHPL and PPL were determined using Lineweaver–Burk plot. The *K*_m_, *k*_cat_ and *k*_cat_/*K*_m_ were 3.6 mM, 1.1 × 10^5^ min^−1^ and 2.9 × 10^4^ for recHPL and 3.9 mM, 1.0 × 10^5^ min^−1^ and 2.7 × 10^4^ for PPL using 2.5–30 mM tributyrin. These values are comparable to the previous result, 5.4 mM, 1.9 × 10^5^ min^−1^ and 3.6 × 10^4^ for native PPL by Gangadhara *et al.* (21).

### Heat and pH stability of recHPL

**Fig. 5:**
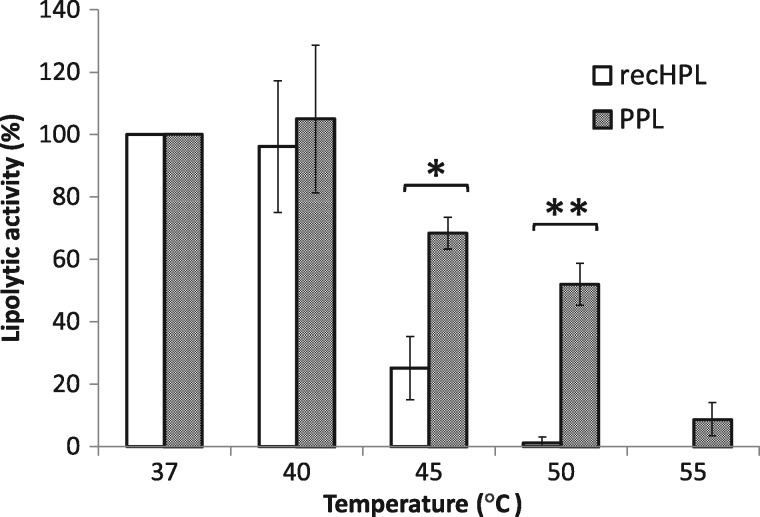
**Heat stability of the lipolytic activity of recHPL and PPL.** Lipolytic activity was measured at the indicated temperatures using the pH-stat technique and tributyrin as substrate. Activities at different temperatures are expressed as a percentage of the activity at 37°C. Data shown represent the mean ± SD values from at least three measurement and were analysed for statistical significance using the Student’s *t* test. *P < 0.05; **P < 0.01.

**Fig. 6:**
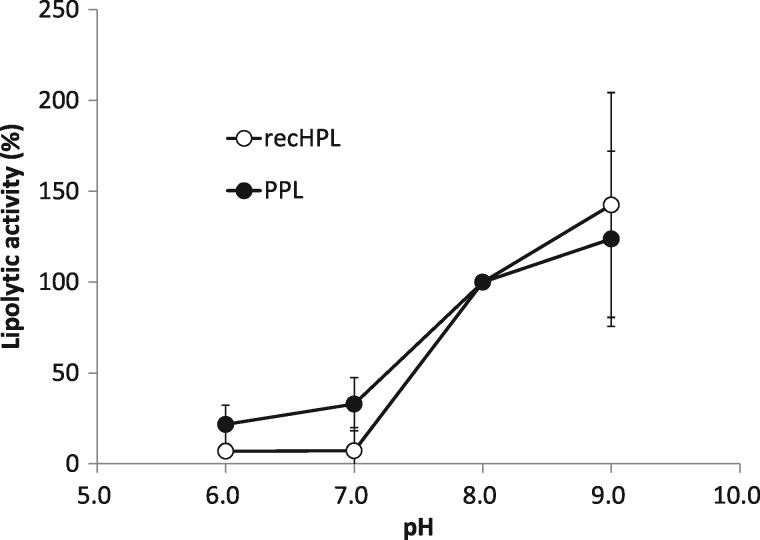
**Dependency of the lipolytic activity of recHPL and PPL on pH.** Activity was measured using pH-stat technique at the indicated pH value at 37°C. Tributyrin was used as substrate. Activities at different pH are expressed as a percentage of the activity at pH 8.0. 1 mM Tris-HCl and 1 mM phosphate buffer were used as buffers at pH 8.0–9.0 and pH 6.0–7.0, respectively. Data shown represent the mean ± SD values from at least three measurements and were analysed for statistical significance using the Student’s *t* test.

The impact of heat and pH on recHPL was measured and compared with the properties of native PPL. Both lipases dissolved in the running buffer for FPLC (10 mM phosphate buffer (pH 8.0), 150 mM NaCl, 400 mM l-Arg) were stable for at least 1 month at 4°C without an observable change in specific activity. PPL and recHPL exhibited the highest lipase activity between 37 and 40°C as shown in Fig. 5. However, recHPL showed much lower lipolytic activity at 45°C and was almost inactivated at 50°C, while PPL retained 50% of its activity at 50°C compared to that at 37°C. As shown in Fig. 6, the activities of recHPL and PPL showed similar pH dependencies in the pH range of 6.0–9.0, with maximum activity observed at pH 8–9.

### Inhibitor sensitivity of recHPL

**Fig. 7:**
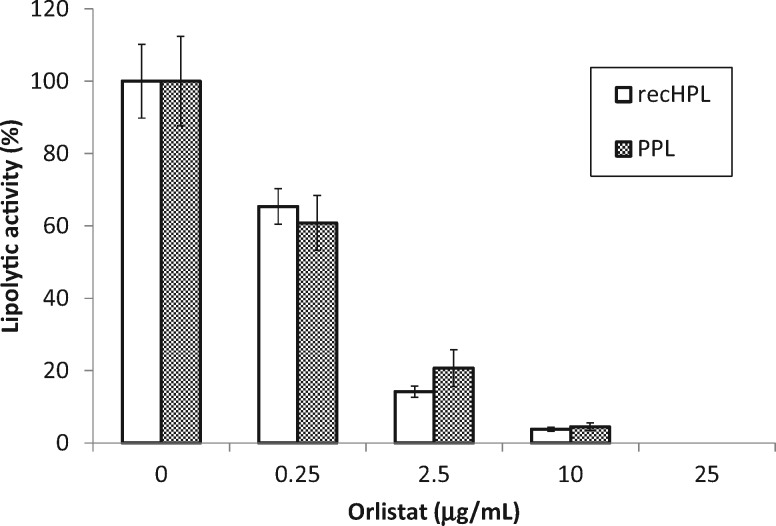
**Sensitivity of recHPL and PPL to Orlistat.** Lipolytic activity was measured at 37°C using the pH-stat technique and tributyrin as the substrate. Activities with different concentrations of Orlistat are expressed as a percentage of the activity. Data shown represent the mean ± SD from at least three measurements.

The inhibitor sensitivity of recHPL was measured using Orlistat and compared with the native PPL. As shown in Fig. 7, both lipases were equally inhibited by each concentration of Orlistat at 37°C.

## Discussion

Our studies show that recHPL purified from *E.coli* had lipolytic activity that was almost equal to the native PPL, and that the optimal temperature and pH for native PPL and recHPL were very similar. Judging by the SDS-PAGE profile of the purified protein under non-reducing conditions, a part of refolded recHPL formed high-molecular weight aggregates consisting of more than 4–5 molecules per aggregate, and we believe that intermolecular disulfide bonds were responsible for this aggregation. Considering that HPL has a large hydrophobic site for adsorption of lipids, it is likely that glycosylation may play a part in preventing the aggregation of HPL molecules *in vivo* due to hydrophobic interaction. We suggest that glycosylation of HPL may therefore contribute to stabilization of the monomer, thus maintaining enzyme activity. Importantly, the presence of l-arginine at 400 mM was found to inhibit the aggregation of recHPL during refolding. Indeed, the amount of soluble recHPL after refolding increased by 3-fold when l-arginine was added to all the dialysis solutions during the refolding steps.

In this study, recHPL was compared to PPL because pigs and humans are closely related species of mammals, and the two pancreatic lipases possess a high sequence homology (86%). Furthermore, PPL has been orally administered for the treatment of human pancreatic exocrine insufficiency (PEI) in humans, which is known as the pancreatic enzyme replacement therapy (PERT) (22), indicating the therapeutic significance of PPL usage for humans.

PERT is a possible application of recHPL after the biological safety of the ST-tagging is demonstrated. The mainstay therapy for PEI is PERT with extracts of porcine pancreas. However, there are no other approved options for the treatment, even though this treatment cannot be used for patients with pig allergies or who have religious objections to consuming pork (22). RecHPL may also be used for screening of inhibitor compounds, which may provide an important avenue for anti-obesity therapy. Other promising applications of recHPL are industrial, because lipases are one of the most widely used biocatalysts for biotechnological processes. Lipases find promising applications in organic chemical processing, detergent formulations, synthesis of biosurfactants and the oleochemical, dairy and agrochemical industries (23, 24).

Although various eukaryotic expression systems have been employed for recHPL such as those using yeast, insect and mammalian cells, the recHPLs obtained using these cells are differentially glycosylated, necessitating stringent quality control, because glycans may alter tissue targeting and half-life of the protein in blood as well as alter the immunogenicity and allergenicity of the recombinant protein. The use of *E.coli* as an expression system can avoid these problems in addition to providing other benefits such as ease of the expression and purification protocol (25). Therefore, in this study, we have attempted to optimize the protocol for expression and purification of HPL in *E.coli*.

Our method of preparing recHPL from *E.coli* has several advantages over those in previous reports where eukaryotic expression systems were utilized. For instance, our protocol is easy to scale up, costs less, and is convenient because it circumvents the need to control the glycosylation pattern of the recombinant protein. Several methods have been reported to express lipase using yeast, insect or mammalian, cells, which require culturing for at least 5, 6 or 10, days, respectively (26–28). Our method requires only 2 days of culturing. Both *E.coli* and yeast expression systems are advantageous due to their low-cost and ease of scaling up, which is most suitable to produce the component of biodetergents. Although the *E.coli* expression system requires refolding, there is no need to control the glycosylation pattern which is required for other three expression systems because the glycosylation may influence enzyme activity and biostability and sometimes may induce unexpected biological interactions such as antigenicity, allergenicity or other inflammatory responses.

## Supplementary Material

Supplementary Figures

## Abbreviations

BSA: bovine serum albumin
CBB: Coomassie Brilliant Blue
CD: circular dichroism
DTNB: 5,5′-dithiobis-2-nitrobenzoic acid
EDTA: ethylenediaminetetraacetic acid
HRP: horseradish peroxidase
LB: Luria-Bertani
PCR: polymerase chain reaction
PL: pancreatic lipase
PMSF: phenylmethylsulfonyl fluoride
PPL: porcine pancreatic lipase
recHPL: recombinant human pancreatic lipase
ST II: Strep-tag II
TBS: 10 mM Tris-buffered saline (pH 7.5)
