# Supplementary material for: A novel protocol for the preparation of active recombinant human pancreatic lipase from Escherichia coli
Source: J Biochem. 2018 Aug 8;164(6):407–14. doi: 10.1093/jb/mvy067 (PMC6267337; doi:10.1093/jb/mvy067)
Supplement: Supplementary Figures [file mvy067_supplementary_figures.pptx]

## Slide 1
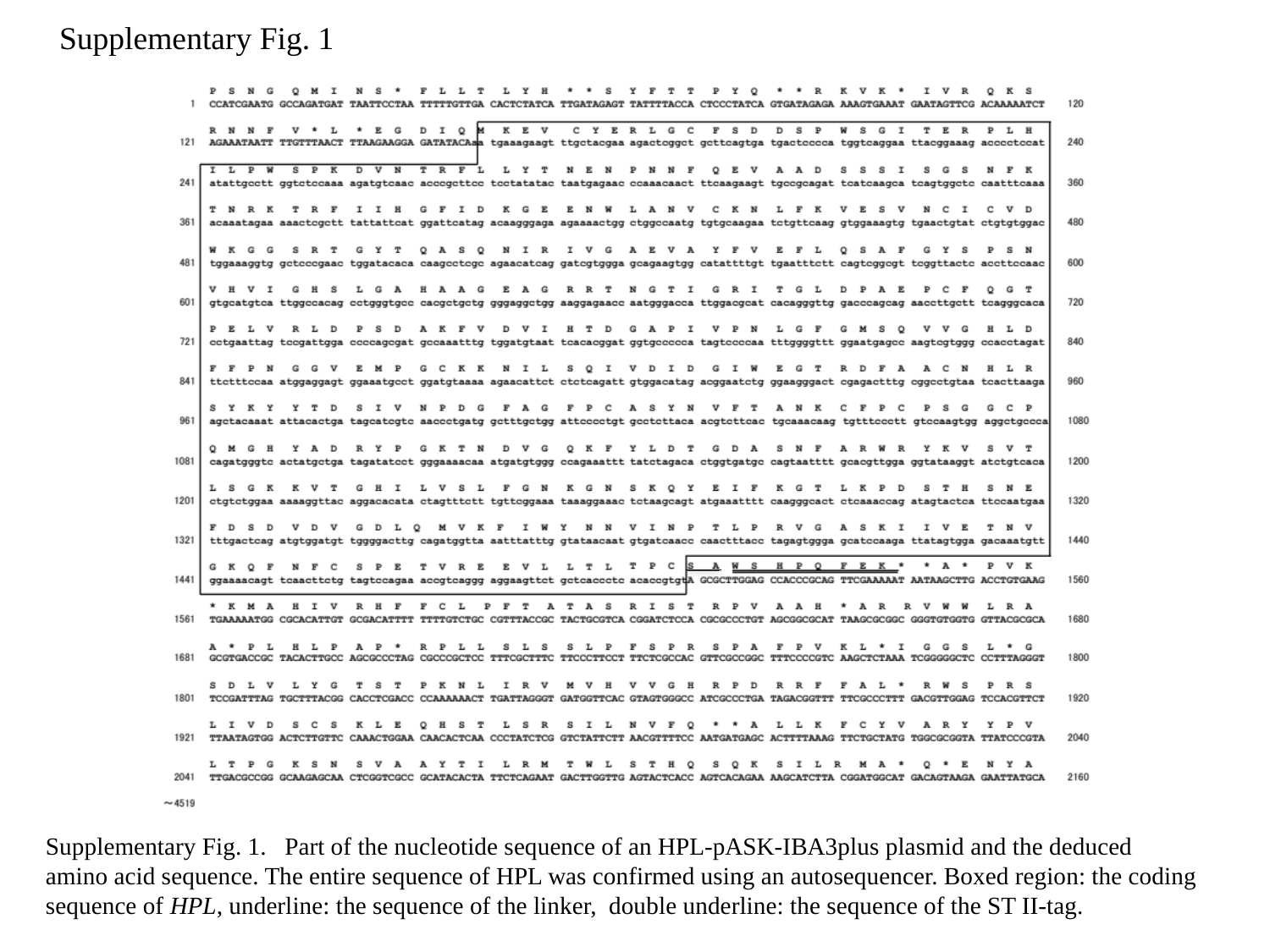

Supplementary Fig. 1
Supplementary Fig. 1. Part of the nucleotide sequence of an HPL-pASK-IBA3plus plasmid and the deduced amino acid sequence. The entire sequence of HPL was confirmed using an autosequencer. Boxed region: the coding sequence of HPL, underline: the sequence of the linker, double underline: the sequence of the ST II-tag.

## Slide 2
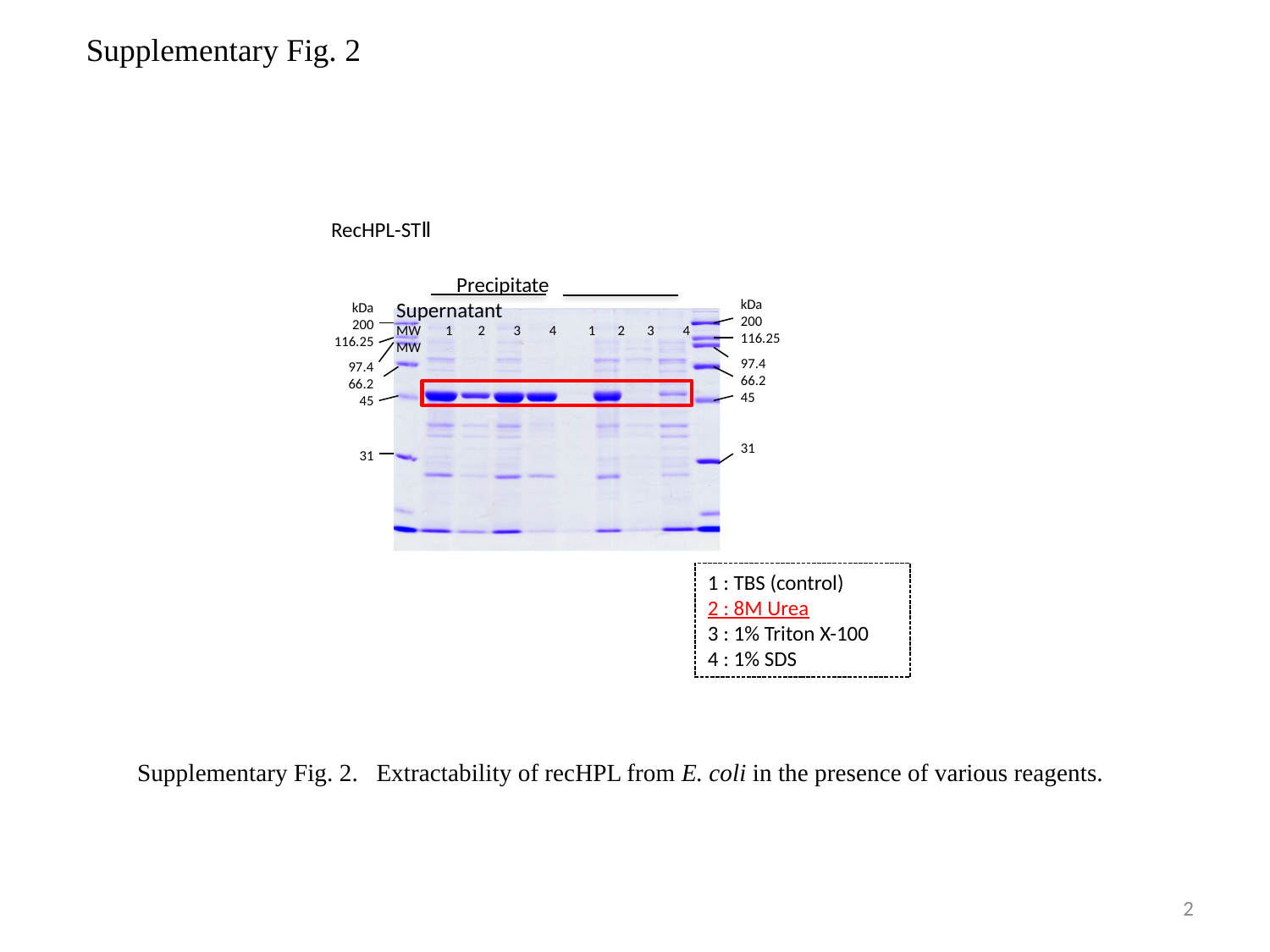

Supplementary Fig. 2
RecHPL-STⅡ
　　　　Precipitate　　 Supernatant
MW　 1 2 3 4 1 2 3 4 MW
kDa
200
116.25
97.4
66.2
45
31
kDa
200
116.25
97.4
66.2
45
31
1 : TBS (control)
2 : 8M Urea
3 : 1% Triton X-100
4 : 1% SDS
Supplementary Fig. 2. Extractability of recHPL from E. coli in the presence of various reagents.
2

## Slide 3
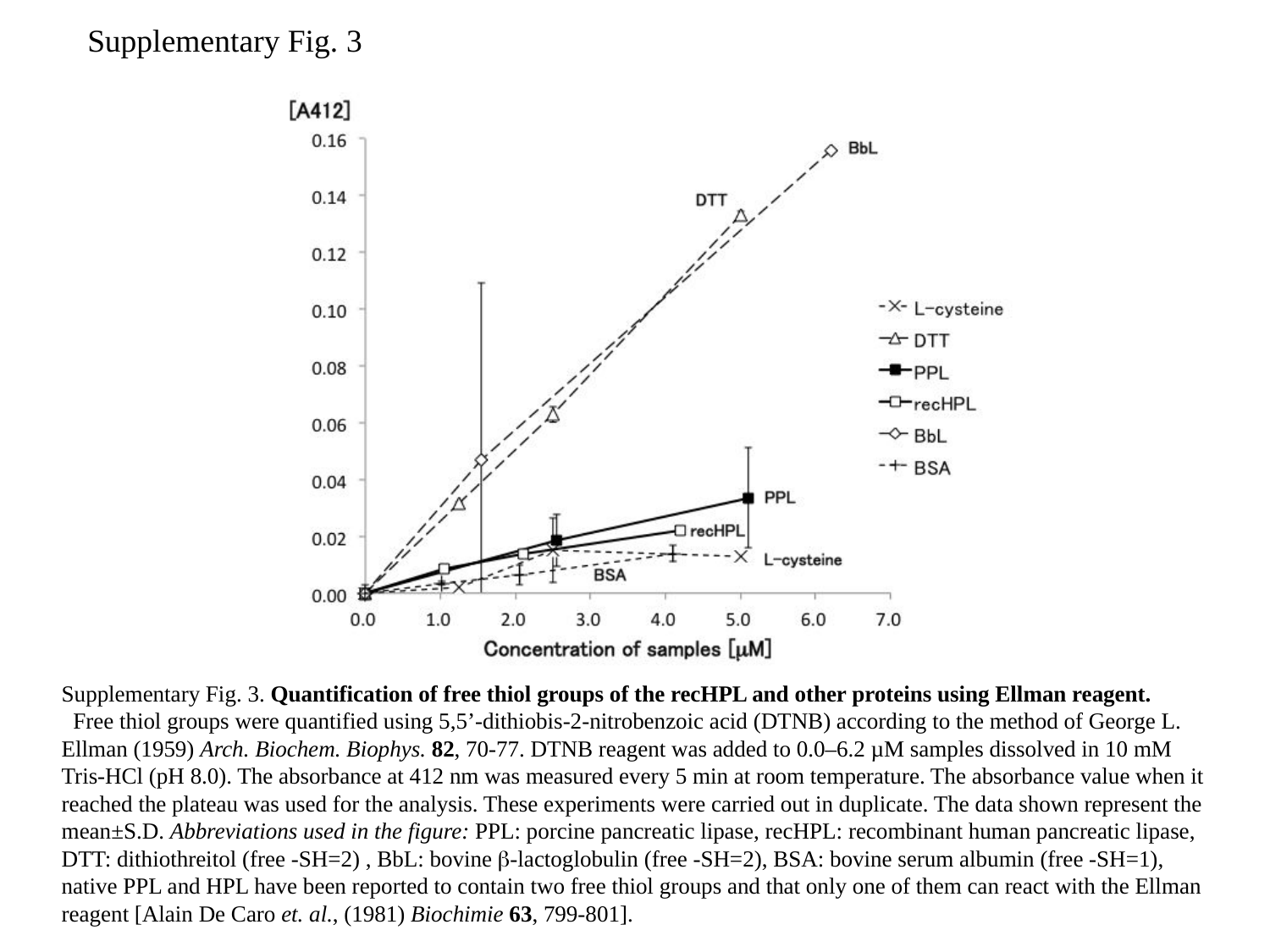

Supplementary Fig. 3
Supplementary Fig. 3. Quantification of free thiol groups of the recHPL and other proteins using Ellman reagent.
 Free thiol groups were quantified using 5,5’-dithiobis-2-nitrobenzoic acid (DTNB) according to the method of George L. Ellman (1959) Arch. Biochem. Biophys. 82, 70-77. DTNB reagent was added to 0.0–6.2 µM samples dissolved in 10 mM Tris-HCl (pH 8.0). The absorbance at 412 nm was measured every 5 min at room temperature. The absorbance value when it reached the plateau was used for the analysis. These experiments were carried out in duplicate. The data shown represent the mean±S.D. Abbreviations used in the figure: PPL: porcine pancreatic lipase, recHPL: recombinant human pancreatic lipase, DTT: dithiothreitol (free -SH=2) , BbL: bovine b-lactoglobulin (free -SH=2), BSA: bovine serum albumin (free -SH=1), native PPL and HPL have been reported to contain two free thiol groups and that only one of them can react with the Ellman reagent [Alain De Caro et. al., (1981) Biochimie 63, 799-801].
